# Epidemiology and molecular characterization of avian influenza A viruses H5N1 and H3N8 subtypes in poultry farms and live bird markets in Bangladesh

**DOI:** 10.1038/s41598-023-33814-8

**Published:** 2023-05-16

**Authors:** Ariful Islam, Shariful Islam, Meerjady S. Flora, Emama Amin, Karlie Woodard, Ashley Webb, Robert G. Webster, Richard J. Webby, Mariette F. Ducatez, Mohammad M. Hassan, Mohamed E. El Zowalaty

**Affiliations:** 1 Centre for Integrative Ecology, School of Life and Environmental Sciences, Deakin University, Geelong, Victoria 3216 Australia; 2 EcoHealth Alliance, New York City, New York 10018 USA; 3 Institute of Epidemiology, Disease Control and Research, Dhaka, 1212 Bangladesh; 4 Division of Virology, Department of Infectious Diseases, St. Jude Children’s Research Hospital, Memphis, Tennessee 38105 USA; 5 Interactions Hôtes-Agents Pathogènes, Institut National de Recherche pour l’Agriculture, l’Alimentation et l’Environnement, Ecole Nationale Vétérinaire de Toulouse, Université de Toulouse, Toulouse, France; 6 Queensland Alliance for One Health Sciences, School of Veterinary Science, The University of Queensland, St Lucia, Queensland 4343 Australia; 7 Faculty of Veterinary Medicine, Chattogram Veterinary and Animal Sciences University, Chattogram, 4225 Bangladesh; 8 Veterinary Medicine and Food Security Research Group, Medical Laboratory Sciences Program, Faculty of Health Sciences, Abu Dhabi Women’s Campus, Higher Colleges of Technology, 41012, Abu Dhabi, UAE

**Keywords:** Microbiology, Virology, Influenza virus, Diseases, Infectious diseases, Influenza virus

## Abstract

Avian influenza virus (AIV) remains a global threat, with waterfowl serving as the primary reservoir from which viruses spread to other hosts. Highly pathogenic avian influenza (HPAI) H5 viruses continue to be a devastating threat to the poultry industry and an incipient threat to humans. A cross-sectional study was conducted in seven districts of Bangladesh to estimate the prevalence and subtypes (H3, H5, and H9) of AIV in poultry and identify underlying risk factors and phylogenetic analysis of AIVs subtypes H5N1 and H3N8. Cloacal and oropharyngeal swab samples were collected from 500 birds in live bird markets (LBMs) and poultry farms. Each bird was sampled by cloacal and oropharyngeal swabbing, and swabs were pooled for further analysis. Pooled samples were analyzed for the influenza A virus (IAV) matrix (M) gene, followed by H5 and H9 molecular subtyping using real-time reverse transcription-polymerase chain reaction (rRT-PCR). Non-H5 and Non-H9 influenza A virus positive samples were sequenced to identify possible subtypes. Selected H5 positive samples were subjected to hemagglutinin (HA) and neuraminidase (NA) gene sequencing. Multivariable logistic regression was used for risk factor analysis. We found that IAV M gene prevalence was 40.20% (95% CI 35.98–44.57), with 52.38%, 46.96%, and 31.11% detected in chicken, waterfowl, and turkey, respectively. Prevalence of H5, H3, and H9 reached 22%, 3.4%, and 6.9%, respectively. Waterfowl had a higher risk of having AIV (AOR: 4.75), and H5 (AOR: 5.71) compared to chicken; more virus was detected in the winter season than in the summer season (AOR: 4.93); dead birds had a higher risk of AIVs and H5 detection than healthy birds, and the odds of H5 detection increased in LBM. All six H5N1 viruses sequenced were clade 2.3.2.1a-R1 viruses circulating since 2015 in poultry and wild birds in Bangladesh. The 12 H3N8 viruses in our study formed two genetic groups that had more similarity to influenza viruses from wild birds in Mongolia and China than to previous H3N8 viruses from Bangladesh. The findings of this study may be used to modify guidelines on AIV control and prevention to account for the identified risk factors that impact their spread.

## Introduction

Influenza A virus is a negative-strand RNA virus belonging to *Orthomyxoviridae* family, and the virion carries surface proteins known as hemagglutinin (HA) and neuraminidase (NA)^1^. Based on their potential to cause disease in chickens, avian influenza viruses (AIVs) are grouped into two categories: highly pathogenic avian influenza virus (HPAIV) and low pathogenic avian influenza virus (LPAIV)^2–4^. AIVs infect chickens, turkeys, and other gallinaceous birds and inflict significant economic losses worldwide^5–7^. In terms of humans and poultry, Bangladesh is one of the world's most densely populated countries. The poultry industry in Bangladesh supports economic growth and poverty reduction in rural and urban areas by creating employment opportunities and food products^8^. LPAIVs and HPAIVs, including the highly pathogenic H5N1 viruses, have been found in waterfowl, pet birds, wild birds, and chickens in Bangladesh^9–13^. In Bangladesh, over 580 outbreaks of HPAI H5N1 have been reported in poultry and wild birds since 2007^14–16^. Because the poultry industry accounts for 20% of the livestock sector in Bangladesh, the culling of an estimated 250 million diseased animals to date in response to these outbreaks causes food insecurity and negatively impacted the economic growth^17^. In addition, eight human cases of H5N1 have been reported in Bangladesh, with one fatality^18^. Human H5N1 infections have been reported in Vietnam, Thailand, Indonesia, Hong Kong, China, and Cambodia, all of which have a history of poultry exposure in LBMs and commercial and free-range farms, implying that both LBMs and farms can contribute to the spread of AIVs among poultry and from poultry to humans^19–22^. Furthermore, the co-circulation of LPAIV is a source of concern.

In Bangladesh, the average number of reported poultry outbreaks caused by AIV subtype H5 per year has declined, dropping from 83 and 10 outbreaks in commercial and backyard poultry, respectively, in 2007–2012 to two and zero outbreaks in 2013–2019. As compensation policies were phased out, underreporting and AIV subtype H5 vaccination in commercial poultry might be among the causes for the reduction in the number of avian influenza outbreaks^23^. LBMs are the backbone of poultry trade in Bangladesh. Birds of different species and geographical origins are introduced into LBMs on daily basis and these birds may be housed together, allowing for local transmission of several virus subtypes and possible reassortment^20,22,24^. On the other hand, commercial and backyard poultry farming contribute to the development of the poultry industry of Bangladesh^25^. Waterfowl, either wild or raised in various settings, such as backyard, nomadic, and free-range, play an additional role in virus transmission among commercial and wild bird populations^26,27^. Most studies aimed at determining the level of viral circulation in poultry are conducted in LBM, with only a few conducted-on poultry farms^24,28–30^. To address this gap and inform AIV control in poultry in both LBMs and farms, a thorough knowledge of infection patterns and risk management is essential. The study presented here quantifies the extent of H5, H9, and H3 virus circulation, factors influencing the spread of AIVs, and phylogenetic analysis of viruses in duck and turkey farms and LBMs in seven representative districts of Bangladesh.

## Results

### Prevalence of influenza A virus M-gene

**Figure 1:**
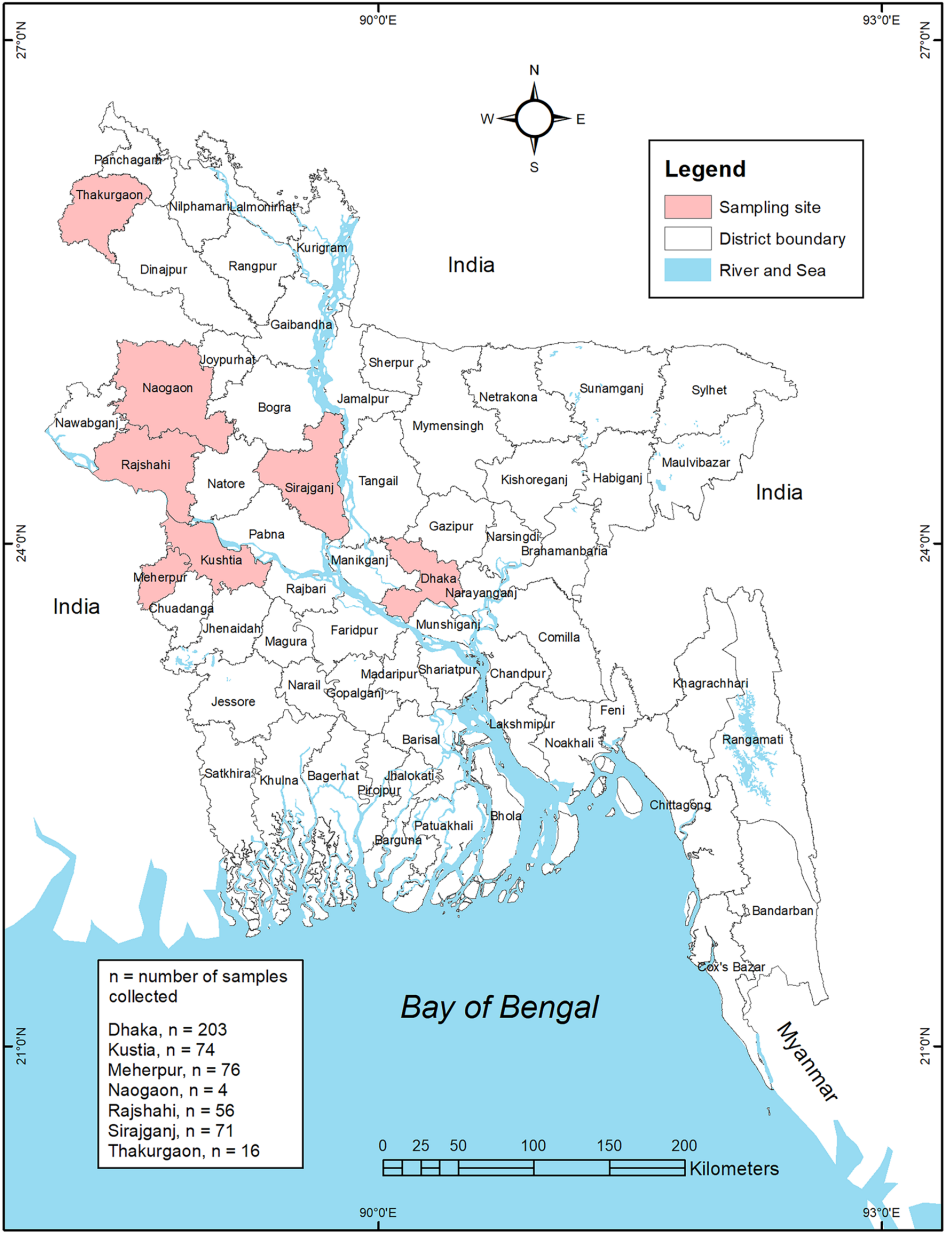
Sample locations of live bird market and poultry farms showing the avian influenza surveillance sites in seven districts in Bangladesh. The map was generated using ArcGIS version 10.4 (http://arcgis.com/).

The prevalence of IAV M-gene was 40% (95% CI 35.98–44.57). In our samples, pigeons had the highest prevalence of IAV/ M-gene (75%, 95% CI 23.68–96.67). Further, 52% of the chicken tested positive for M-gene (95% CI 31.78–72.19), and 47% (95% CI 40.80–53.21) of waterfowl were M-gene positive. In addition, IAV M-gene was detected in 31% of the turkey swabs (Fig. 1).

### Overall prevalence of IAV subtypes (H3, H5, and H9)

**Figure 2:**
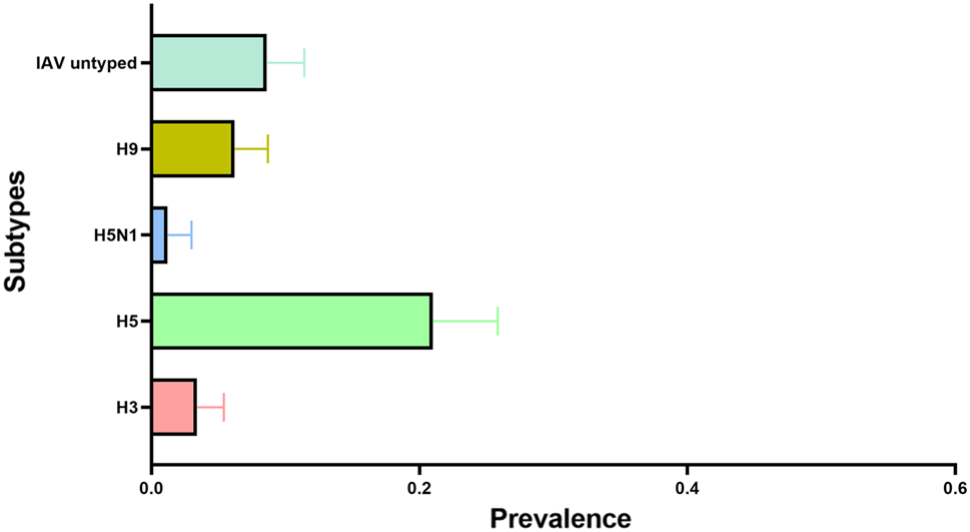
The prevalence (including 95% binomial confidence intervals) of AIV subtypes  H3, H5, H9, and untyped (other subtypes) AIV.

Figure 2 presents the prevalence of influenza A virus subtypes. IAV subtype H5 had the highest prevalence (22%, 95% CI 18.58–25.85). The prevalence of IAV subtype H3 and IAV subtype H9 was 3.4% and 6.9%, respectively.

### Prevalence of IAV subtypes (H3, H5, H9) in host taxa

**Figure 3:**
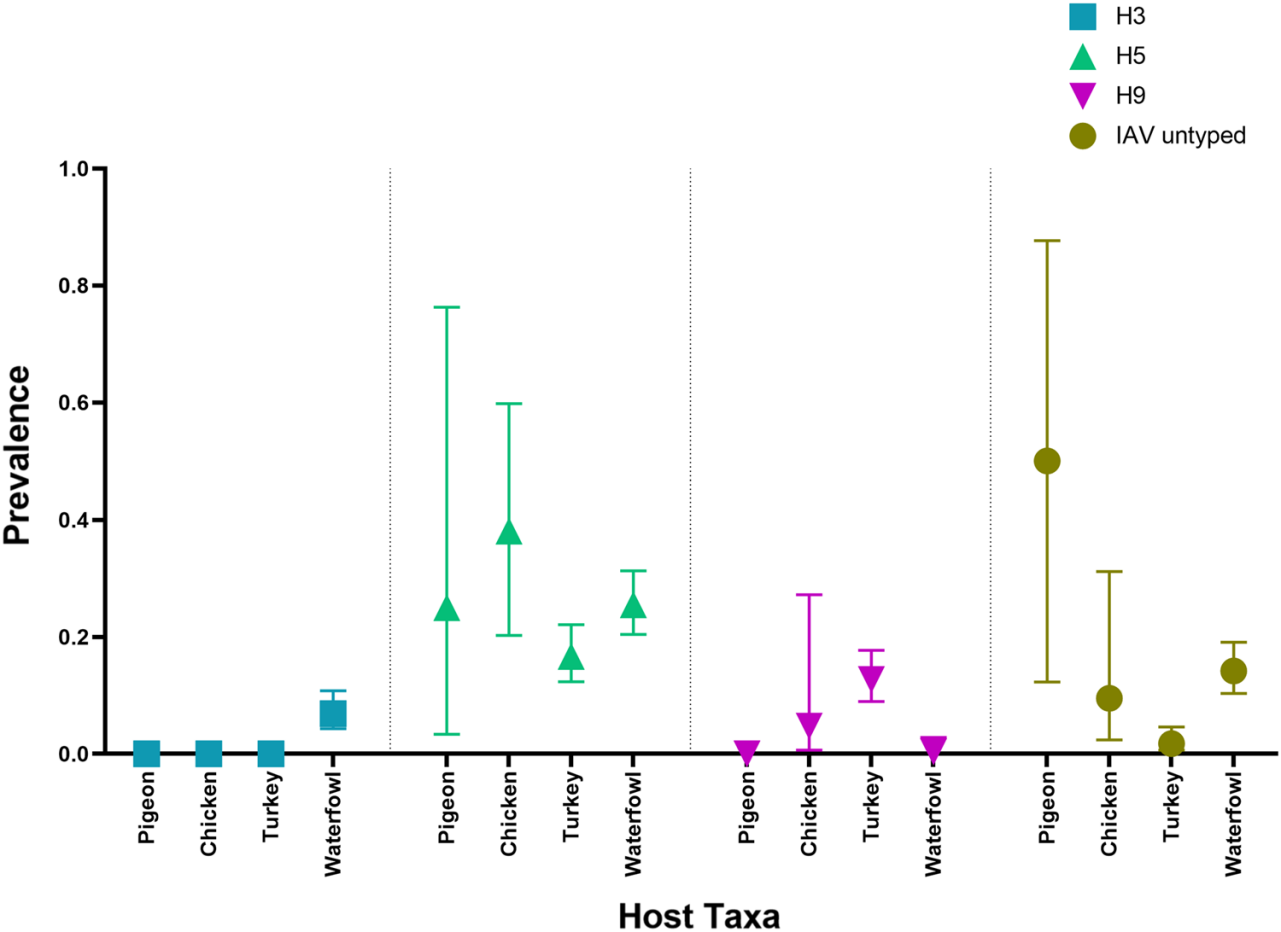
Prevalence (including 95% binomial confidence intervals) of AIV subtypes H3, H5, H9 and IAV untyped across different poultry species and waterfowl.

IAV subtype H3 was detected only in waterfowl (6.88%, 95% CI 4.31–10.80). On the other hand, IAV subtype H5 was detected in all the species. The prevalence of subtype H5 was highest in chickens (38.09%, 95% CI 20.29–59.81), while 25.51% (95% CI 20.45–31.32) of the waterfowl tested positive for IAV subtype H5. Further, 25% of pigeons and 16% of turkeys were found to have IAV subtype H5. However, IAV subtype H9 was not detected in waterfowl. Only 12.71% of the turkey and 4.76% of the chicken samples were positive for IAV subtype H9, as shown in Fig. 3.

### Prevalence of IAV subtypes (H3, H5, and H9) in the LBM-Farm interface

**Figure 4:**
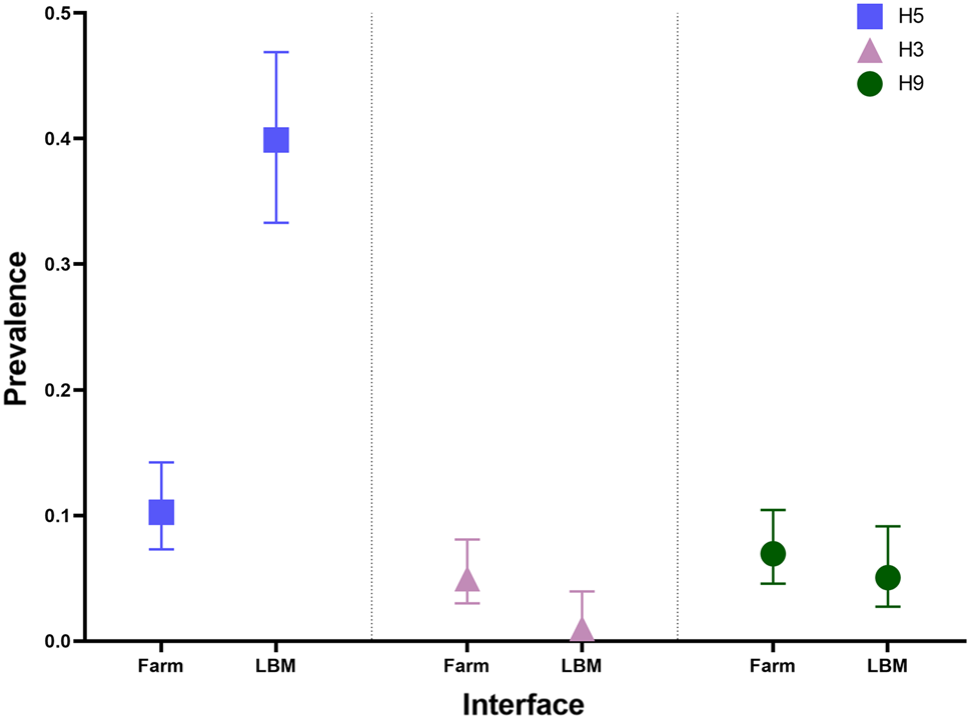
Prevalence of AIV subtypes H3, H5, and H9 at poultry farm and LBM interface.

The prevalence IAV subtype H5 was much higher in LBMs (39.9%; 95% CI 33.29–46.89) than in farms (10.3%; 95% CI 7.30–14.28). However, the farms had a higher prevalence of both H3 (Farm: 4.97%, LBM: 1.01%) and H9 (Farm: 6.95%, LBM: 5.05%) viruses than LBM, as shown in Fig. 4.

### Choropleth map of the prevalence (IAV subtypes H5, H3, and H9) by the district

**Figure 5:**
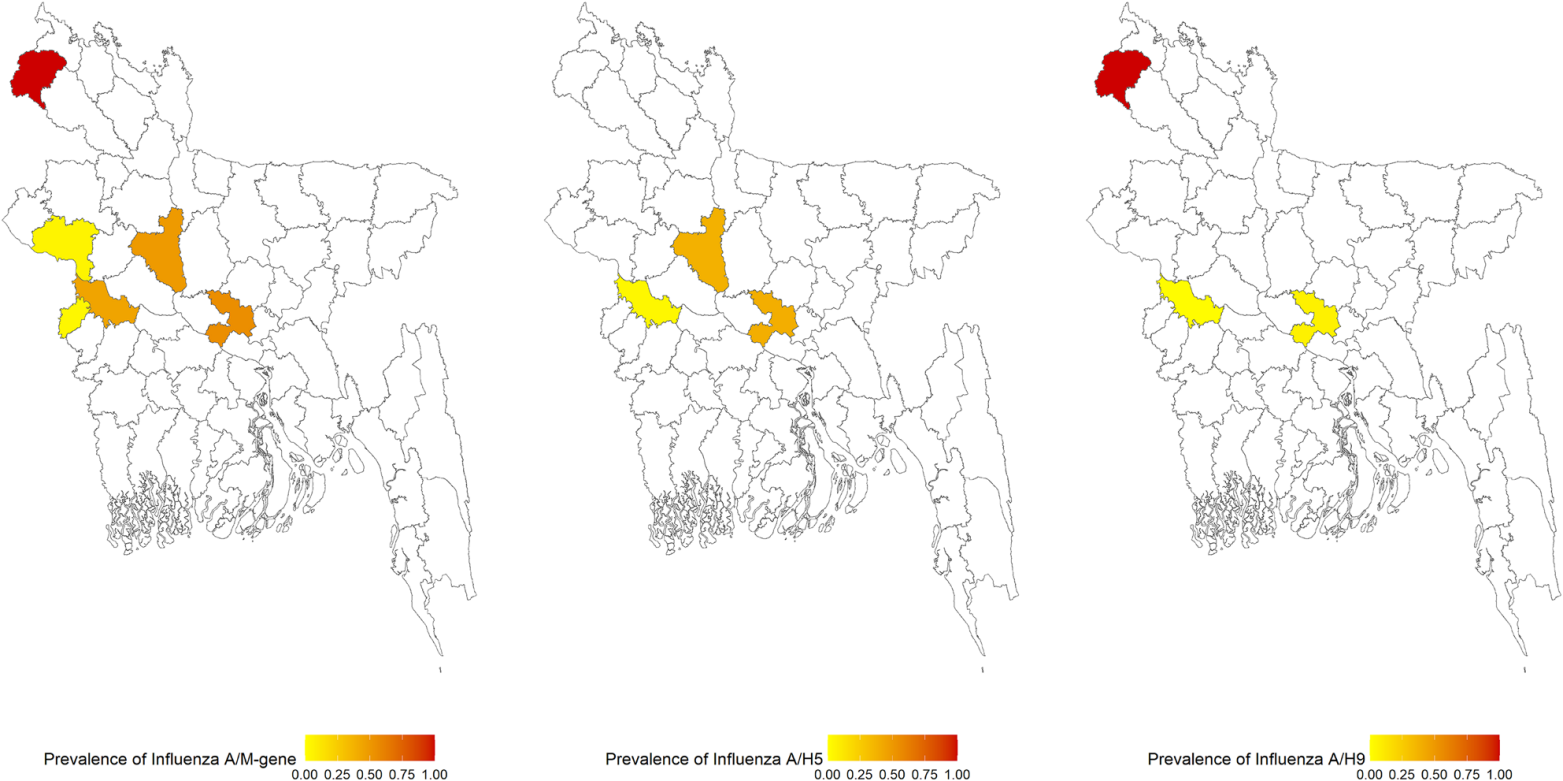
Choropleth map of the prevalence of avian influenza A virus M-gene, H5, and H9 subtypes identified from the samples in different districts. The map was generated using RStudio version 4.1.2.

The prevalence of IAV M-gene was highest in Thakurgaon. All the samples collected from Thakurgaon were positive for IAV M and subtype H9 (Fig. 5). The prevalence of IAV M-gene was 55% and 49% in Dhaka and Sirajganj, respectively. IAV subtype H5 was detected in 39% and 38% of the samples from Dhaka and Sirajganj, respectively. While the prevalence of IAV subtype H9 was 7% in Dhaka, 0.99% and 1.41% of the samples were IAV subtype H3 positive in Dhaka and Sirajganj, respectively. The prevalence of the IAV M-gene was 38% and was 17.57% for IAV subtype H3 in Kushtia. However, a very low prevalence was recorded for IAV subtypes H5 (4%) and H9 (1%). The prevalence of IAV M-gene was very low in Meherpur (4%) and Rajshahi (5%). In addition, no samples from Rajshahi and Meherpur were detected as being H5 positive. All samples from Naogaon tested negative for the M-gene.

### Bivariate analysis to determine the factors associated with IAV M-gene and IAV subtype H5

Bivariate analyses were conducted to determine the factors associated with M-gene and H5 positivity. The results of the bivariate analysis are given in Table 1. Season, host taxa, health status, and interface were considered. Results from the chi-square test suggest that all the variables were significant risk factors for M-gene and subtype H5 (at 5% significance level). Prevalence was higher in winter than in summer for M-gene (71.11%, 95% CI 60.92–79.54) and subtype H5 (41.11%, 95% CI 31.43–51.54). Based on the interface, the prevalence of M-gene was higher in LBM (53.54%) than in farms (31.46%). The same was true for subtype H5.

**Table 1:** Factors associated with IAV M-gene and H5 subtype (Prevalence and Chi-square test).

Variable	Category	Influenza A virus M-gene			Influenza A virus subtype H5		
		% (n)	95% CI	*P*-value	% (n)	95% CI	*P*-value
Season	Summer	33.41 (137)	(29, 38.14)	< 0.001	17.8 (73)	(14.39, 21.82)	< 0.001
	Winter	71.11 (64)	(60.92, 79.54)		41.11 (37)	(31.43, 51.54)	
Host Taxa	Pigeon	75 (3)	(23.68, 96.67)	< 0.001	25 (1)	(3.33, 76.32)	0.033
	Chicken	52.38 (11)	(31.8, 72.19)		38.1 (8)	(20.29, 59.81)	
	Turkey	31.14 (71)	(25.45, 37.46)		16.67 (38)	(12.36, 22.09)	
	Waterfowl	46.96 (116)	(40.8, 53.22)		25.51 (63)	(20.45, 31.32)	
Health status	Dead	73.68 (56)	(62.66, 82.37)	< 0.001	61.84 (47)	(50.47, 72.05)	< 0.001
	Healthy	34.2 (145)	(29.83, 38.86)		14.86 (63)	(11.77, 18.58)	
Interface	Farm	31.46 (95)	(26.46, 36.93)	< 0.001	10.26 (31)	(7.31, 14.24)	< 0.001
	LBM	53.54 (106)	(46.55, 60.39)		39.9 (79)	(33.3, 46.89)	

### Modeling

The multivariable logistic regression modeling of M-gene suggests that season, host taxa, and health status significantly affect M-gene prevalence. However, the season is not a significant factor for subtype H5, but host taxa, health status, and interface are significant factors. The results of the multivariable logistic regression analysis are shown in Table 2. In winter, the chances of being positive for M-gene were 4.93 times higher than in summer, and waterfowl had 4.75 times the odds of being positive for M-gene and 5.71 times the odds of being positive for subtype H5 than chicken.

**Table 2:** Determinants of IAV M-gene and H5 subtype (results from multivariable logistic regression).

Variable	Category	Influenza A virus M-gene		Influenza A virus subtype H5	
		AOR	95% CI	AOR	95% CI
Season	Summer	1		1	
	Winter	4.93 **	(2.75, 8.85)	1.65	(0.9, 3.06)
Host taxa	Chicken	1		1	
	Pigeon	3.23	(0.33, 43.81)	0.61	(0.05, 7.01)
	Turkey	2.11	(0.63, 7.0468)	3.02	(0.95,9.59)
	waterfowl	4.75**	(1.47, 15.38)	5.71**	(1.88, 17.37)
Health status	Healthy	1		1	
	Dead	5.76**	(2.45, 13.52)	6.92**	(3.20, 14.94)
Interface	Farm	1		1	
	LBM	1.15	(0.68, 1.95)	3.28**	(1.75, 6.14)

**indicates *P*-value < 0.05.

In terms of health, dead birds were substantially more likely than healthy birds to be both M-gene positive and subtype H5 positive. Furthermore, a bird from LBM had a 3.28 times higher chance of being subtype H5 positive than a bird from a farm (Table 2).

### Phylogenetic analysis of HA and NA genes of H5N1 and H3N8

#### H5N1

**Figure 6:**
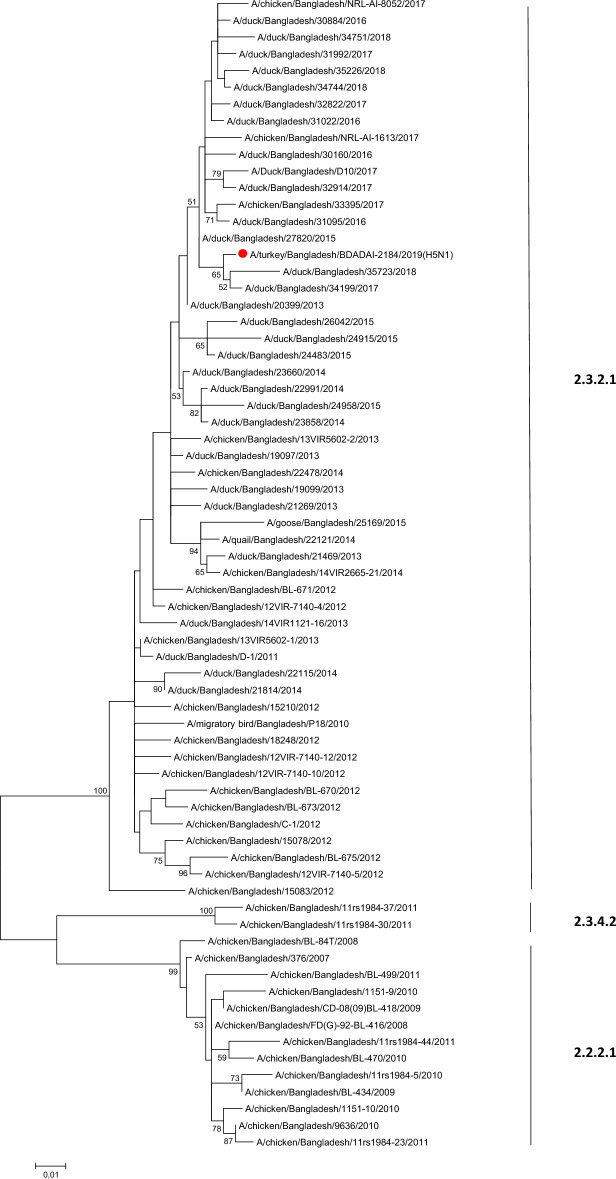
Phylogenetic analysis of HA gene of H5N1 viruses. Maximum Likelihood tree (HKY + G model) with 500 boostraps (values > 50 shown on branches only); the sequence of the present study was highlighted with a red closed circle. As all H5 sequences from the present study were identical, only A/turkey/Bangladesh/BDADAI-2184/2019 was kept as representative strain. H5 clades are indicated on the right-hand side of the tree.

Phylogenetic analyses of HA and NA genes of all six H5N1 viruses were performed. H5N1 viruses have evolved into multiple clades including  2.2.2, 2.3.4.2, 2.3.2.1c, and 2.3.2.1a. All the six characterized viruses in the present study, were collected in 2019 (two from Dhaka, three from Kushtia, and one from Sirajganj), shared identical HA and NA sequences and clustered with newly reassorted clade 2.3.2.1a sequences from Bangladesh (Fig. 6 and Supplementary Fig. 1). Four substitutions known to play a role in increased alpha-2,6 sialic acid receptor binding have been identified in the HA sequences of the H5N1 strains isolated in the current study: D94N, S155N, T156A and K189R (H5 numbering). No classical neuraminidase inhibitor resistance markers were detected^31^.

#### H3N8

**Figure 7:**
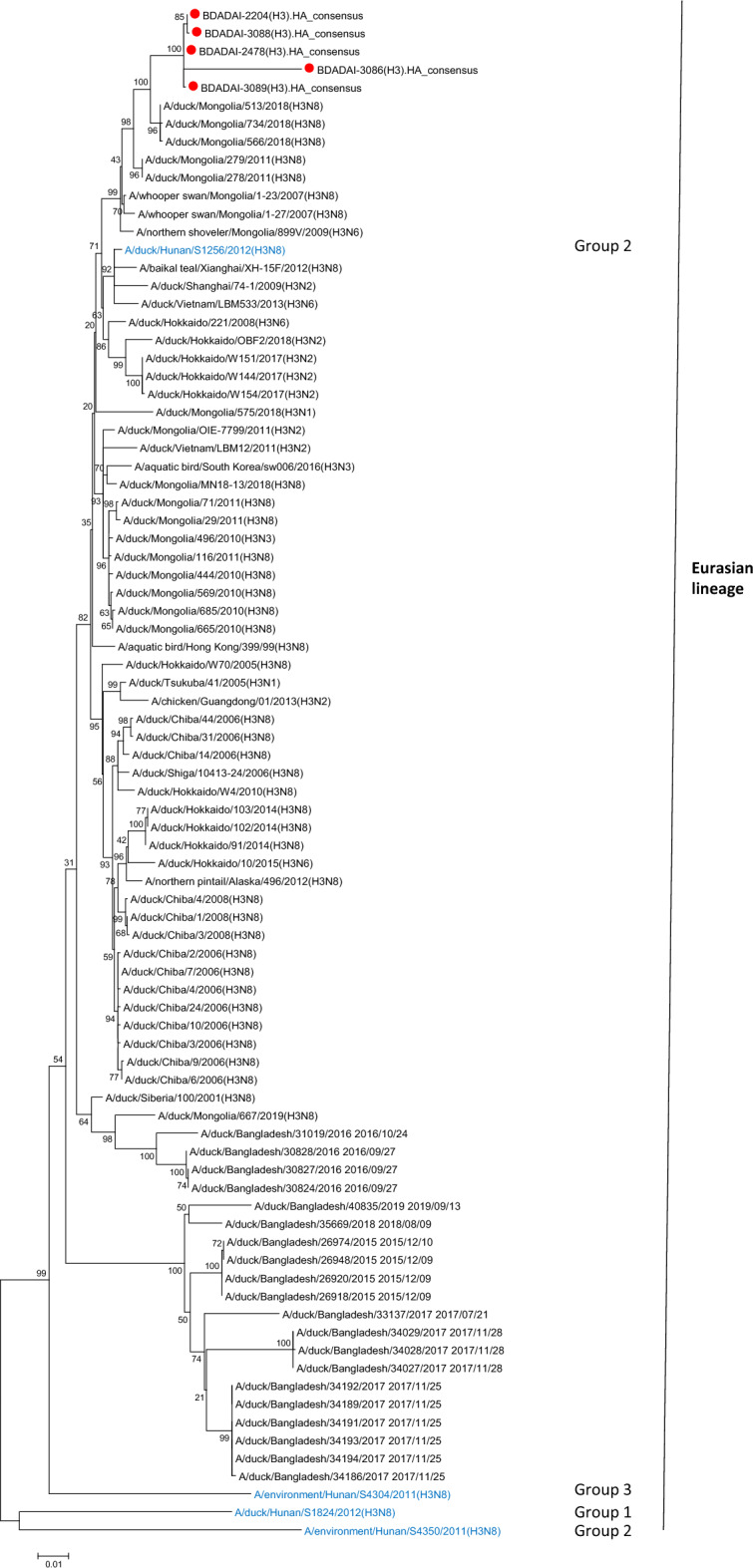
Phylogenetic analysis of HA gene of H3N8 viruses. Maximum Likelihood tree (HKY + G model) with 500 boostraps (values > 50 shown on branches only); the sequences of the present study were highlighted with a red closed circle. As four H3 sequences from the present study were identical, only A/duck/Bangladesh/BDADAI-2204/2019 was kept as representative of A/duck/Bangladesh/BDADAI-2561/2019, A/duck/Bangladesh/BDADAI-3147/2019, and A/duck/Bangladesh/BDADAI-3237/2019. H3 genotypes (as defined in reference 53) are indicated on the right-hand side of the tree. Reference sequences are in blue text.

Phylogenetic analyses of HA and NA segments were also performed to gain better understanding of the evolutionary relationships between the characterized 12 H3N8 viruses (eight from Kushtia, two from Dhaka, one from Rajshahi, and one from Sirajganj). The HA segments of the twelve H3 viruses were grouped into the Eurasian lineage (Fig. 7). Ten H3 viruses were identical and clustered in one group; the other two viruses were identical and clustered in another group. The HA sequences of the twelve H3 viruses were genetically more similar to sequences of viruses from Mongolia, China, and Japan than the previously reported H3 viruses from Bangladesh. The twelve N8 neuraminidase genes grouped into the Eurasian lineage. The N8 sequences were closely related to those of viruses from Mongolia and China (Supplementary Fig. 2).

## Discussion

AIVs including highly pathogenic strains, can cause zoonotic disease and are often associated with severe economic, animal, and public health consequences^32^. Influenza A viruses (IAVs) subtype H5 have raised significant concern worldwide due to their high pathogenicity and zoonotic potential. More concerningly, three human infections with avian influenza A (H3N8) virus were reported from China as of April 2023^33^. As a result, determination of the variables that impact influenza virus prevalence is essential. This study investigated the risk factors associated with influenza A, specifically H5 subtype prevalence. The present study detected influenza A viruses in waterfowl, chicken, turkey, and pigeon, similar to previously reported data^34–36^. We screened avian samples and found evidence of H3, H5, and H9 viruses in the tested samples, albeit with specific patterns. No H7 subtype viruses were detected in the tested samples. IAVs subtype H9 were detected only in turkey and chicken samples, and were not detected in waterfowl samples. A higher prevalence of H9 in chickens than waterfowl in LBM was previously reported^37,38^ similar to the present study. The prevalence of IAV subtype H3 in the present study was lower than that reported in other South Asian countries^39^. We detected IAV H3 subtype only in waterfowl, which is consistent with the finding that H3 viruses are among the most commonly identified subtypes in ducks (proportion up to 91.76%)^38,39^. In addition, previous IAVs H3 subtype from Bangladesh were detected primarily in ducks^41,42^. IAV subtype H3 was detected in Kushtia, Sirajganj, Rajshahi, and Dhaka in the present study and more often in farms than LBMs, suggesting wild bird involvement through interactions with free-range farmed ducks.

In the present study, IAV subtype H5 had the highest prevalence. IAV subtype H5 has been spreading in Bangladesh since 2007, with more than 500 outbreaks reported in chickens. Analysis showed that all IAV subtype H5 in the tested samples belonged to the 2.3.2.1a clade. It was also found that the prevalence of influenza A M-gene and influenza A subtypeH5 were greater in chickens than in waterfowls, but the logistic regression model showed an increased risk of the IAV M-gene and IAV subtype H5 in waterfowls, similar to a previous report^43^.

Results of the current study showed that the risk of AIVs was higher in the winter than in the summer, consistent with many outbreaks in Bangladesh and other countries occurring during the same season^35,44–46^. This may be explained due to low temperatures and low humidity, which favor AIVs persistence^47^. The odds of IAV H5 subtype detection were higher in LBM than in farms in the present study. The LBM biosecurity in Bangladesh is widely seen as falling short of minimum acceptable standards, and their capacity to act as viral evolution drivers facilitating the genesis of new emerging strains is significant^48^.

Molecular markers of influenza HA and NA are associated with increased virulence, adaptation to mammals, or resistance to antiviral agents. Unfortunately, no information on avian H3N8 virus molecular markers is available in literature, except for mutation W222L in HA was shown to allow for equine to dog adaptation for influenza A H3N8^49^. However, many molecular markers HA and NA are well characterized for H5N1. In the present study, four substitutions D94N, S155N, T156A and K189R (H5 numbering) which were previously reported to play a role in the increased α -2,6 SA receptor binding have been identified in the HA of influenza A H5N1 in Bangladesh and no classical neuraminidase inhibitor resistance markers were detected in the NA sequences.

Among the limitations of the present study is that only six H5N1 and twelve H3N8 viruses were selected for sequencing and only HA and NA were sequenced. This limited our ability to conduct accurate spatial and temporal analyses of the data. Detailed sequence analysis of the internal gene segments of the tested H5 viruses may add significant data regarding whether these viruses are similar to previously circulating AIVs in the sub-continent or whether these viruses are further evolving by reassortment with other subtypes. However, sequencing of internal gene segments of the tested  influenza viruses in the present study was not possible due to lack of resources.

The findings of the present study could also be extended to include biosecurity practices such as disposal of dead bird, handling of sick bird, and other factors influencing AIV transmissibility and prevalence.

In conclusion, the findings of the present study highlight that multiple subtypes of AIVs (H5, H3, and H9) are circulating in both LBM and farms in Bangladesh. We identified the season, host type, and health status of birds as risk factors influencing AIV prevalence in the selected study areas. Farmers and workers in poultry farms are highly encouraged to get trained regularly to prevent possible AIV zoonotic transmission. The present study shows that avian influenza viruses are circulating in poultry populations in LBMs in Bangladesh, emphasizing the need for more extensive genomic surveillance studies to determine the avian influenza gene pool, monitor the possibility of emergence of new viruses of zoonotic potential, and to track their transmission. Routine and programed surveillance will help the early detection and rapid responses to prevent possible AI outbreaks. Many developing countries have similar poultry rearing practices in LBMs and farms as in Bangladesh. As a result, such recommendations based on our data and findings have global significance and implications.

## Materials and methods

### Sample collection

We conducted a cross-sectional study at the LBM and farm interfaces. In the instance of LBM, only LBMs in Dhaka, the capital and largest city in Bangladesh, were sampled due to the large numbers and concentration of LBMs and the presence of an AIV gene pool. On the other hand, for farms, the majority of live birds in Dhaka originate from the country's northeast part. In light of this fact, we collected duck and turkey samples from farms in seven major districts in the northeastern part of the country. Cloacal and oropharyngeal swab samples were collected from chickens (*Gallus gallus domesticus*), domestic ducks (*Anas platyrhynchos domesticus)* pigeons (*Columba livia domestica*) and turkey (*Meleagris gallopavo*) birds between September 2018 and November 2019 from seven districts, as shown in Fig. 1, using sterile swabs which were placed in a 1.8 mL sterile cryo-vial containing 1 mL of viral transport media (VTM) as previously described^49^.

### RNA extraction and AIV rRT-PCR

Pooled cloacal and oropharyngeal samples (n = 500) were shipped to the Division of Virology, Department of Infectious Diseases, St. Jude Children’s Research Hospital (Memphis, TN, USA) for virus isolation and characterization. We extracted the viral RNA using the RNeasy Mini Kit (Qiagen, USA), and cDNA was synthesized using the SuperScript™ III Reverse Transcriptase (Invitrogen, USA). We tested all swab samples using real-time reverse transcription PCR (rRT-PCR) using universal M gene and H5, H3, H7 and H9 HA-specific primers, as previously described^51,52^. We considered a positive result for any gene tested if the Ct (cycle threshold) was less than 35 and with a characteristic amplification curve.

### Virus isolation

Influenza A virus M-gene positive samples were cultured for virus isolation by inoculation in embryonated chicken eggs (ECEs) as previously described^32^. A volume of 100 μl from each swab sample was injected into the allantoic fluid (AF) of three 10-day-old ECEs per sample and incubated at 35°C. After 72 hr, the AF was collected and tested for hemagglutination using 0.5% turkey erythrocytes. All the first passage in ECE (E1) AF samples were tested using the Flu Detect® (Zoetis Inc.) and RNA was extracted from AF samples and was retested for AIV by M gene rRT-PCR. The AIV-positive RNA were submitted for sequencing.

### DNA sequencing

Influenza A virus positive samples were submitted to Hartwell Sequencing Center facility, St. Jude Children's Research Hospital, for sequencing using Illumina Techniques (Illumina, CA, USA). M-gene positive, H5/H9 negative samples were subtyped using molecular methods using DNA sequencing. A multisegment RT-PCR- was performed using gene-specific primers according to the previously published protocol to amplify the whole influenza viral genome^53^. PCR products were then gel-extracted and purified using GE Healthcare illustra™ GFX PCR DNA and Gel Band Purification Kit (Sigma Aldrich, MO, USA). Library preparation of samples was performed using Illumina's Nextera XT DNA Sample Preparation kit (Illumina, CA, USA) according to the manufacturer's protocol. Amplicons were sequenced on the Illumina's MiSeq platform using the paired-end approach. Sequencing reads were demultiplexed, quality-trimmed and filtered prior to consensus sequence generation using the Pallas pipeline, developed by the Hartwell Genomics Centre at St. Jude Children's Research Hospital. Final analysis and the generation of consensus sequences were completed using CLC Genomics Workbench (v.11.0.1).

### Phylogenetic analysis and molecular characteristics

Multiple sequence alignment with the MUSCLE algorithm and phylogenetic and evolutionary analyses were conducted using IQ-Tree^54^ and MEGA11^55^. Briefly, phylogenetic trees of full-length HA and NA gene segments of six H5N1 viruses and 12 H3N8 viruses were generated using the maximum likelihood method with Tamura–Nei model and 1000 bootstraps replicate. All HA and NA sequences of gene segments of AIVs subtype H5N1 from Bangladesh available in the https://www.ncbi.nlm.nih.gov/genbank/ and in the EpiFlu database of the Global Initiative on Sharing All Influenza Data https://gisaid.org/ were retrieved and used as references, together with the first 50 blast hits for each gene segment and subtype, and for H3 and N8 trees together with reference sequences from the known genotypes as previously described^56^. In addition to H5N1 viruses from Bangladesh, the phylogenetic analyses also included H5N1 viruses from other countries with the closest genetic similarity, as indicated by the BLAST search. The phylogenetic analyses comprised the 12 H3N8 viruses from Bangladesh and other countries which were most closely related to H3 viruses for HA and N8 viruses for NA, according to the BLAST search. The HA and NA gene trees included sequences of representative Eurasian and North American H3 subtype viruses from thehttps://www.ncbi.nlm.nih.gov/genbank/ and https://gisaid.org/ databases.

### Statistical analysis

#### Data entry and cleaning

We recorded both field and laboratory data in Microsoft Excel 2016. The data set was cleaned, coded, recorded, and checked for integrity in Microsoft Excel before exporting to R (Version 4.1.1, RStudio) for data analysis.

#### Exploratory analysis

We performed descriptive analysis to calculate M gene, H3, H5, and H9 prevalence based on rRT-PCR and sequencing results. The prevalence of M gene, H3, H5, and H9 were also calculated by each host taxa. To visualize the prevalence, graphs with error bars and confidence intervals were used. We used the software R version 4.1.2 and R studio program (Version 4.1106, Integrated Development for R. RStudio, PBC, MA, USA) and GraphPad Prism (Version 8.0.2) (GraphPad Software, Inc., CA, USA). We prepared the choropleth maps depicting the prevalence of IAV H5, H9, and H3 subtypes by districts using R (Version 4.1.1, RStudio).

#### Bivariate analysis and statistical modeling

Bivariate analysis was conducted for different factors related to IAV M-gene and H5 in the study area. Seasons were classified into temporal patterns (winter and summer). Host taxa were categorized into pigeon, turkey, chicken, and waterfowl. Health status was classified based on healthy and dead birds. The interface was divided into LBM and Farm. “Chi-Square Test” was performed using R to identify the association of factors with the prevalence of the IAV M-gene and H5. Variables that had a *p*-value < 0.05 were considered for further statistical modeling. For statistical modeling, we utilized significant variables in the bivariate analysis. As our outcome variables on IAV M-gene, and IAV subtype H5 were binary, we considered multivariable logistic regression for each outcome.

### Ethical approval

All procedures and methods were carried out in accordance with relevant guidelines and regulations and were reported in accordance with ARRIVE guidelines. All procedures were approved by the Ethics Committee of the Chattogram Veterinary and Animal Sciences University, Bangladesh, under reference number CVASU/Dir (R&E) EC/2019/126/(1).

## Supplementary Information

Supplementary Figure 1.

Supplementary Figure 2.

Supplementary Legends.

## Data Availability

Sequence data generated in the present study were deposited in GenBank, National Library of Medicine, NCBI under accession numbers OK081811, OK081812, OK087622, OK087623, OK087625, OK087624, OK087633, OK087634, OK087635, OK087636, OL375219, OL375220, OL375221, OL375222, OL375223, OL375224, OL375226, OL375227, OL375234, OL375236, OL375237, OL375238, OL376360, OL376361, OL376362, OL376424, OL376425, ON755037, ON755038, ON755058, ON755123, ON755190, and ON755191. In addition, sequences were deposited in the Global Initiative on Sharing All Influenza Data https://www.gisaid.org/ under accession numbers EPI1887774, EPI1887775, EPI1888008, EPI1888009, EPI1888010, EPI1888011, EPI1888012, EPI1888013, EPI1888014, EPI1888015, EPI1888336, EPI1888337, EPI1888338, EPI1888339, EPI1888340, EPI1888341, EPI1888342, EPI1888343, EPI1889089, EPI1889090, EPI1889091, EPI1889092, EPI1889093, EPI1889094, EPI1889095, EPI1889096, EPI1889097, EPI1889098, EPI1889099, EPI1889100, EPI1889101, EPI1889102, EPI1889103, EPI1889104, EPI1889105, and EPI1889106.
