# Supplementary figures and images for: Epidemiology and molecular characterization of avian influenza A viruses H5N1 and H3N8 subtypes in poultry farms and live bird markets in Bangladesh
Source: Sci Rep. 2023 May 16;13:7912. doi: 10.1038/s41598-023-33814-8 (PMC10188517; doi:10.1038/s41598-023-33814-8)

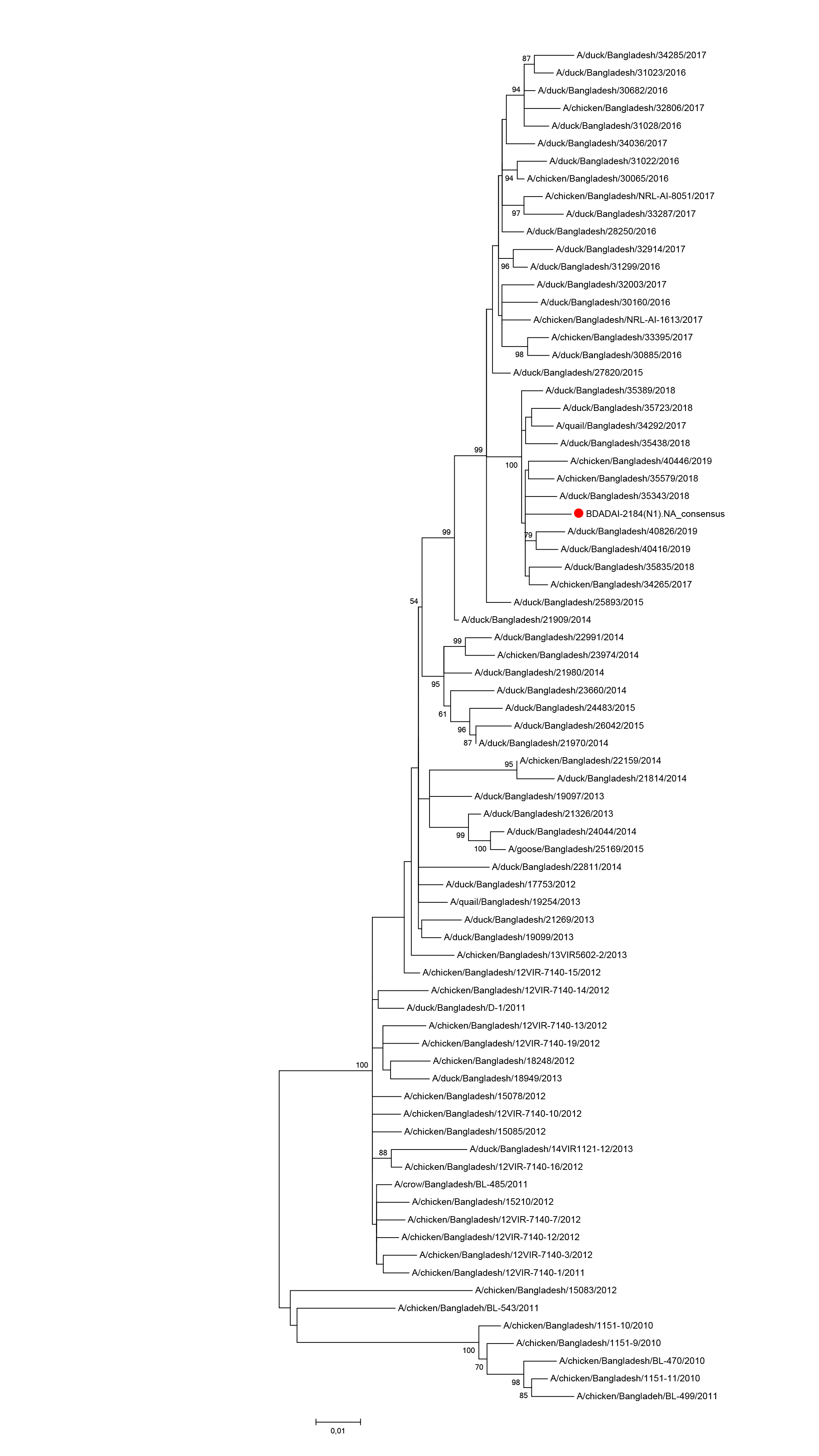

Supplement: Supplementary file 1 — Supplementary Figure 1. [file 41598_2023_33814_MOESM1_ESM.png]

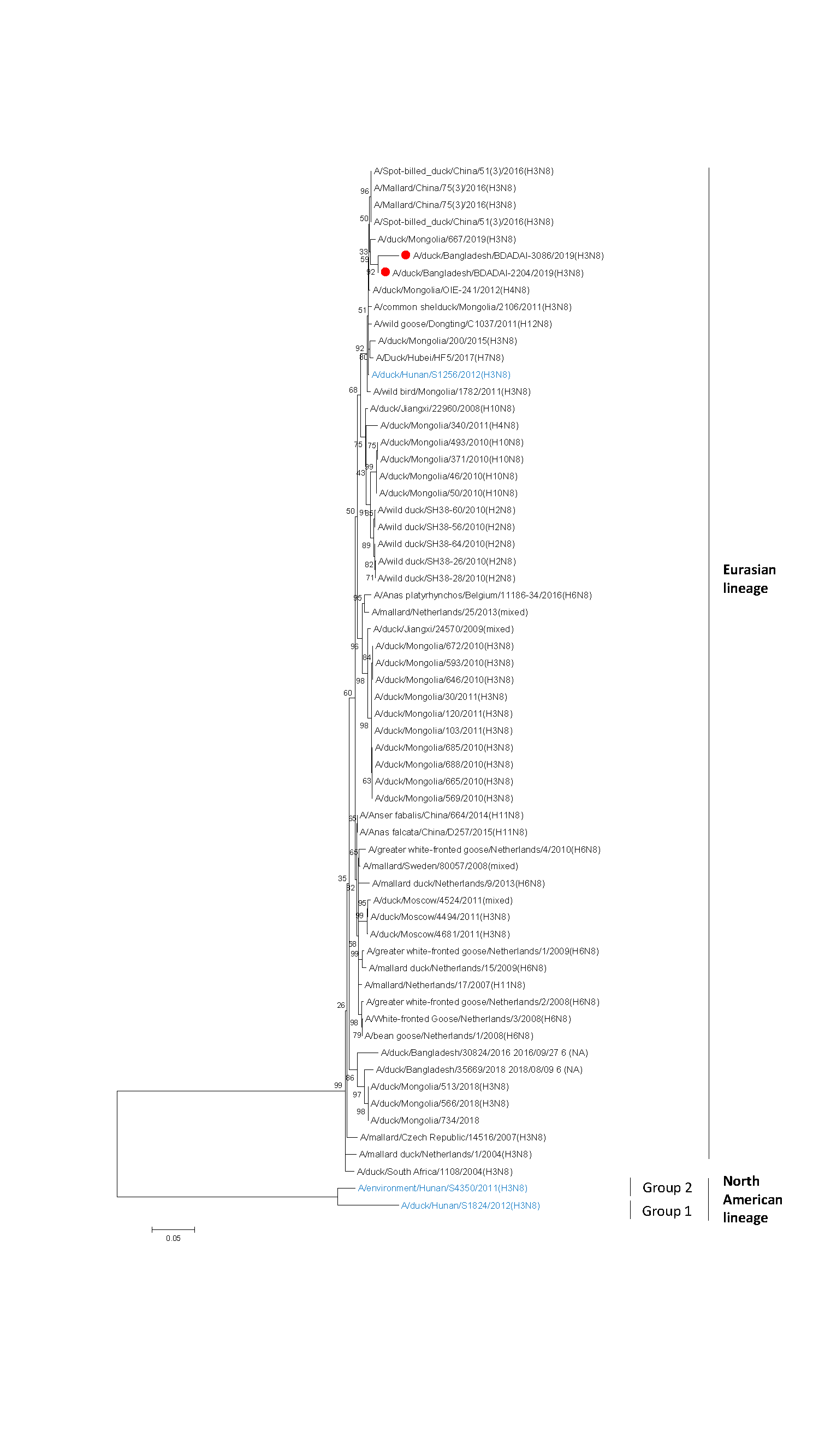

Supplement: Supplementary file 2 — Supplementary Figure 2. [file 41598_2023_33814_MOESM2_ESM.png]
