# Supplementary material for: Epidemiology and molecular characterization of avian influenza A viruses H5N1 and H3N8 subtypes in poultry farms and live bird markets in Bangladesh
Source: Sci Rep. 2023 May 16;13:7912. doi: 10.1038/s41598-023-33814-8 (PMC10188517; doi:10.1038/s41598-023-33814-8)
Supplement: Supplementary file 3 — Supplementary Legends. [file 41598_2023_33814_MOESM3_ESM.docx]

**Supplementary Figure 1:** Phylogenetic analysis of NA gene of H5N1 viruses. Maximum Likelihood tree (HKY+G model) with 500 boostraps (values >50 shown on branches only); the sequence of the present study was highlighted with a red closed circle. As all N1 sequences from the present study were identical, only A/turkey/Bangladesh/BDADAI-2184/2019 was kept as representative strain.

**Supplementary Figure 2:** Phylogenetic analysis of NA gene of H3N8 viruses. Maximum Likelihood tree (HKY+G model) with 500 boostraps (values >50 shown on branches only); the sequences of the present study were highlighted with a red closed circle. As all N8 sequences from the present study were identical to A/duck/Bangladesh/BDADAI-2204/2019 strain except A/duck/Bangladesh/BDADAI-3086/2019, only A/duck/Bangladesh/BDADAI-2204/2019 and A/duck/Bangladesh/BDADAI-3086/2019were kept as representative strains. N8 genotypes (as defined in reference 53) are indicated on the right-hand side of the tree. Reference sequences are in blue font.
